# Cuproptosis-related Gene Signatures and Immunological Characterization in Sepsis-associated Acute Lung Injury

**DOI:** 10.2174/0113862073290692240509094709

**Published:** 2024-05-22

**Authors:** Mingyu Zhu, Xiaokai Tang, Jingjing Xu, Yuanqi Gong

**Affiliations:** 1 Department of Intensive Care Unit, The Second Affiliated Hospital, Jiangxi Medical College, Nanchang University, Nanchang, Jiangxi 330006, China;; 2 Department of Orthopaedic, The First Affiliated Hospital, Jiangxi Medical College, Nanchang University, Nanchang, Jiangxi 330006, China

**Keywords:** Sepsis, acute lung injury, cuproptosis, immunity, signature, bioinformatics

## Abstract

**Background:**

Sepsis is a frequent cause of Acute Lung Injury (ALI), characterized by immune dysregulation and a high mortality rate. The role of cuproptosis, a recently discovered cell death mechanism, in sepsis-associated ALI is still unclear. The study aimed to investigate the regulatory mechanisms and immune characteristics associated with cuproptosis in sepsis-associated ALI, with implications for novel diagnostic and therapeutic approaches.

**Methods:**

Data from the GEO database was utilized to conduct a comprehensive analysis of the cuproptosis-related genes (CRGs) in sepsis-associated ALI. Gene enrichment analysis, WGCNA, CIBERSORT algorithm, and consensus clustering were employed to investigate the associations between CRGs and immune cells. A predictive model for sepsis-associated ALI was developed based on key CRGs, and its diagnostic accuracy was assessed. Finally, qPCR was employed to validate alterations in the expression of CRGs in the sepsis-associated ALI cellular model.

**Results:**

A total of 14 CRGs were identified in sepsis-associated ALI. Strong correlations between the CRGs and immune cells were observed, and two different CRG subtypes were identified. The expression of immune-related factors in both the CRG and gene clusters exhibited similarities, suggesting a connection between the subgroups and immune cells. The prediction model effectively forecasted the incidence of sepsis-associated ALI based on the expression of CRGs. Finally, qPCR analysis confirmed that the expressions of CRGs in the sepsis-associated ALI cell model closely matched those identified through bioinformatic analyses.

**Conclusion:**

The study comprehensively evaluated the complex relationship between cuproptosis and sepsis-associated ALI. CRGs were found to be significantly associated with the occurrence, immune characteristics, and biological processes of sepsis-associated ALI. These findings provide valuable new insights into the mechanisms underlying sepsis-associated ALI.

## 1 INTRODUCTION

Sepsis, a life-threatening condition, is defined as organ dysfunction caused by a maladaptive response to infection [1]. This global health issue affects millions each year, with a high mortality rate, making it a leading cause of death in ICU [2]. Acute lung injury (ALI) and acute respiratory distress syndrome (ARDS) are characterized by lung conditions often linked to sepsis [3]. These conditions are common and severe manifestations of multiple organ dysfunction syndrome in sepsis [1]. Current treatment options include anti-inflammatory drugs, lung-protective ventilation, and conservative fluid therapy [4]. Despite these treatments, sepsis-associated ALI has a high mortality risk and lacks specific pharmacotherapies [5]. Understanding the molecular mechanisms underlying ALI is crucial for identifying treatment targets for sepsis-associated ALI [6].

A novel form of cell death, termed “cuproptosis,” has been identified as copper-dependent [7]. Copper is an essential cofactor for all organisms, but exceeding the threshold maintained by the body's homeostatic mechanisms can lead to toxicity [8]. Cuproptosis results in protein aggregation, proteotoxic stress, and cell death by copper binding to lipoylated enzymes in the Tricarboxylic Acid (TCA) cycle [7]. FDX1 facilitates lipoylation and aggregation of enzymes involved in the mitochondrial TCA cycle, such as DBT, GCSH, DLST, and DLAT, during cuproptosis [9]. SLC31A1 and ATP7B, as copper importers and exporters, respectively, influence the sensitivity of cuproptosis and impact cellular function [10, 11]. Additionally, molecules like LIAS, LIPT1, DLD, DLAT, PDHA1, PDHB, MTF1, GLS, and CDKN2A have been linked to cuproptosis and may play a role in immune regulation [12, 13]. Studies have associated cuproptosis with various diseases, including cancer [14], Alzheimer's disease [15], heart disease [16], and inflammatory diseases [17]. Modulation of cuproptosis by changes in immune cell infiltration can guide individualized immunotherapy [18]. Inflammatory cytokines can induce lung injury, and understanding immune regulatory mechanisms in ALI is crucial for early prevention, diagnosis, and treatment [19, 20]. The role of Cuproptosis-Related Genes (CRGs) in sepsis-associated ALI remains unclear, highlighting the need to explore the relationship between CRGs and ALI.

This study is the first to explore the role of CRGs in sepsis-associated ALI, evaluating the differential expression in sepsis-associated ALI. Through bioinformatics analyses, the gene expression profiles of sepsis-associated ALI were investigated, revealing correlations between CRGs, risk models, and the immune microenvironment. Hub genes related to the clustering of CRGs were identified using the Weighted Gene Co-expression Network Analysis (WGCNA) algorithm, and enrichment analysis was performed to understand the functional characteristics of CRGs. A nomogram was developed for accurate risk prediction in patients with sepsis-associated ALI. Our findings provide novel insights into the progression and treatment of sepsis-associated ALI.

## 2 MATERIALS AND METHODS

### 2.1 Data Sources and Analysis Tools

A search was conducted in the GEO database (www.ncbi.nlm.nih.gov/geo/) using “sepsis” and “sepsis-associated ALI” as keywords to identify gene expression profiling datasets in the peripheral blood of patients. The GSE65682 dataset was based on the platform GPL13667, and patients selected from this dataset were divided into two groups: a sepsis-associated ALI group (n=192) and a healthy control group (n=42). For gene validation in the nomogram, the GSE95233 and GSE66890 datasets were utilized based on platforms GPL570 and GPL6244, respectively. The GSE95233 dataset consisted of 51 septic patients and 22 healthy controls, while the GSE66890 dataset included 32 sepsis cases and 18 sepsis-associated ALI cases.

### 2.2 Analysis of CRG Expression

A total of 52 CRGs were collected for bioinformatics analysis from relevant studies published in the last two years [8, 12, 21, 22]. Perl software was utilized to extract gene symbol information from the platform file, convert the gene probe matrix into a gene expression matrix, and annotate the genes in the dataset [23]. Subsequently, gene expression data from the sepsis-associated ALI group and the control group were extracted using the “limma” R package, followed by automatic log2 transformation for data normalization [24]. Target CRGs expression matrix was extracted from the normalized data, and differential expression analysis between the groups was conducted using the “limma” R package, with cut-off values set at log2 (fold change) > 2 and adjusted *P <* 0.05 [25]. The correlations between CRGs in sepsis-associated ALI were explored using the “corrplot” R package.

### 2.3 Evaluation of the Correlation with Immune Cells

The proportions of immune cell types in relation to the CRG expression were analyzed using ssGSEA and CIBERSORT. ssGSEA calculates the enrichment scores of immune cell-associated genes in the sample gene sets with the “GSVA” and “GSEABase” R packages, incorporating immune-enhancing and immune-suppressing cells [26, 27]. The CIBERSORT algorithm (https://cibersort.stanford.edu/) was utilized to estimate the relative abundance of immune cells in each sample [28]. The expression and enrichment of immune cells, along with the distribution of pro-inflammatory cytokines, were evaluated across different groups.

### 2.4 Consistency Clustering Identification and Analysis

Unsupervised clustering analysis was conducted to categorize sepsis-associated ALI samples based on differentially expressed CRGs using the “ConsensusClusterPlus” R package [29]. We first removed the controls and kept only data from sepsis-associated ALI samples. Clusters K ranging from 2 to 9 were explored to determine the optimal number of subtypes for cluster analysis. Subsequently, Principal Component Analysis (PCA) was utilized to visualize the clustering results, while Gene Set Variation Analysis (GSVA) was employed to assess the enrichment of various metabolic pathways across cluster subgroups [30]. “GSEABase” and “GSVA” R packages were utilized to assess gene enrichment in subgroups associated with metabolic pathways.

### 2.5 Analysis of Biological Functions Among Subtypes

The “WGCNA” R package was used to perform WGCNA to identify co-expressed genes among all expressed genes in the samples and detect co-expression modules significantly associated with CRG clusters [31]. Initially, the samples were clustered with a power index ranging from 1 to 20. Subsequently, the optimal power value was determined by calculating the fitting index and average connectivity and transforming the adjacency relationships into a Topological Overlap Matrix (TOM). Following this, genes were clustered to identify dynamic modules, which were then clustered to identify similarities between them. The module with the lowest p-value in the correlation analysis results was selected as the hub gene for CRG subtypes.

The hub genes from modules strongly associated with the CRG subtypes were chosen for further analysis through Gene Ontology (GO) and Kyoto Encyclopedia of Genes and Genomes (KEGG) enrichment analyses. GO enrichment categories include biological processes (BP), Cellular Components (CC), and Molecular Functions (MF) [32]. KEGG enrichment analysis can help identify important signaling pathways related to Biological Processes [33]. The analyses utilized the “org.Hs.eg.db”, “DOSE,” “clusterProfiler,” and “enrichplot” R packages to match genes with those enriched in relevant pathways.

### 2.6 Construction and Validation of the Machine Learning Model

The machine learning model was developed to evaluate the significance of CRGs in sepsis-associated ALI by analyzing the differential expression of these genes. The expression of the target CRGs in sepsis-associated ALI was extracted to construct four machine learning models, including Generalized Linear Model (GLM), Random Forest (RF), Support Vector Machine (SVM), and Extreme Gradient Boosting (XGB), which were built using the R packages “cart,” “DALEX,” “randomForest,” “kernlab”, “pROC”, and “xgboost” [34]. The accuracy of the model was assessed through the reverse cumulative distribution of residuals, a box line plot of the residuals, and the receiver operating characteristic (ROC) curves. Gene importance was analyzed across the four methods, with the top five CRGs based on importance scores identified. The five disease signature CRGs from the most accurate model were further analyzed. Nomograms were created using “rms” and “rmda” R packages to score the selected disease-signature genes and predict the probability of a patient having the disease based on gene scores [35]. Calibration curves, decision curve analysis, and ROC curves on datasets GSE66890 and GSE95233 were used to validate the accuracy and predictive value of the nomogram scoring.

### 2.7 Cells and Cell Culture

The human alveolar epithelial cell A549 was obtained from Procell (Wuhan, China) and authenticated using short tandem repeat (STR) profiling. The A549 cells were cultured in F-12K medium supplemented with 10% fetal bovine serum and 1% penicillin and streptomycin at 37 °C with 5% CO_2_. Cells were treated with 10μg/ml lipopolysaccharide (LPS) (Sigma-Aldrich, St Louis, MO, USA) for 24 hours in preparation for subsequent qPCR analysis.

### 2.8 Quantitative Real-Time PCR

Total RNA was extracted from the cells using the MolPure Cell/Tissue Total RNA Kit (Yeasen, China), and its concentration and purity were measured with a NanodropC spectrophotometer (Thermo Fisher, Waltham, MA, USA). Subsequently, the RNA (1000 ng) was reverse-transcribed to cDNA using the Hifair III 1st Strand cDNA Synthesis Kit (Yeasen). For qPCR analysis, Hieff UNICON Universal Blue qPCR SYBR Green Master Mix (Yeasen) was utilized on an ABI Prism 7900HT Fast Real-Time PCR system (Thermo Fisher). The amplification process included an initial denaturation step at 95 °C for 2 min, followed by 40 cycles at 95 °C for 10 s and 60°C for 30 s. The relative mRNA expression levels were calculated using the 2^−ΔΔCT^ method, with the housekeeping gene GAPDH used as an internal reference. With the housekeeping gene GAPDH serving as an internal reference. The primer sequences used in the experiments can be found in Table **1**.

### 2.9 Statistical Analysis

The bioinformatics analysis tools utilized in this study included Strawberry Perl (version 5.30.1) for efficient text processing and R language (version 4.1.3) for comprehensive system analysis and visualization. Specific data analysis packages within R were described in the corresponding methods sections. Statistical analysis of qPCR results was performed using GraphPad Prism software (version 9.5.0), with significance set at p-values <0.05.

## 3 RESULTS

### 3.1 Data Preprocessing and Identification of CRGs in Sepsis-associated ALI

**Fig. (1):**
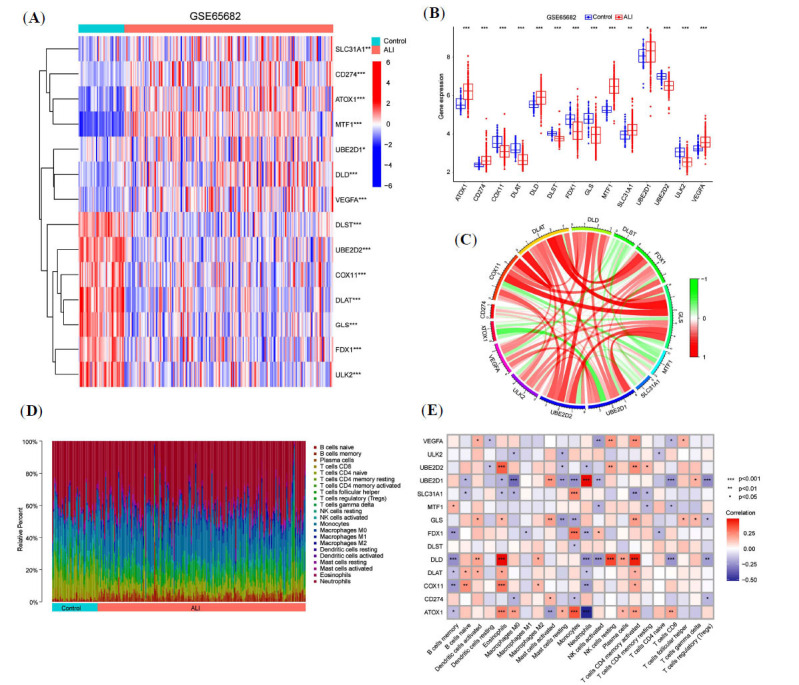
Distribution and relevance of CRGs in sepsis-associated ALI. (**A**) Heatmap showing the expression of the 14 key CRGs in healthy controls and sepsis-associated ALI patients. (**B**) The expression levels of 14 CRGs in the healthy control group and sepsis-associated ALI patients. (**C**) Correlations between CRGs in sepsis-associated ALI patients. (**D**) The relative abundance of infiltrating immune cells between healthy controls and sepsis-associated ALI patients. (**E**) Correlation analysis between 14 differentially expressed CRGs and infiltrating immune cells. *P <0.05, **P < 0.01, ***P < 0.001.

A total of 14 CRGs with significant differences were identified in the sepsis-associated ALI dataset (Fig. **1A**). The expression of SLC31A1, CD274, ATOX1, MTF1, UBE2D1, DLD, and VEGFA was found to be upregulated, while that of DLST, UBE2D2, COX11, DLAT, GLS, FDX1, and ULK2 were significantly downregulated in patients with sepsis-associated ALI (Fig. **1B**). Subsequently, the relationships between these CRGs were explored through correlation analysis (Fig. **1C**). The results revealed significant positive correlations between GLS and DLAT as well as COX11 and a significant positive correlation between FDX1 and DLAT. Moreover, a significant negative correlation was observed between ATOX1 and UBE2D1 expression.

The study further analyzed immune cell infiltration in sepsis-associated ALI compared to healthy controls. Results indicated a higher presence of memory B cells, plasma cells, γδT cells, monocytes, M0 macrophages, and activated mast cells in sepsis-associated ALI (Fig. **1D**). Correlation analysis between 14 CRGs and immune cells in sepsis-associated ALI showed that DLD was significantly positively correlated with eosinophils, resting NK cells, and activated CD4+ memory T cells. In contrast, UBE2D1 was significantly positively correlated with neutrophils and negatively correlated with M0 macrophages, while ATOX1 showed a significant negative correlation with neutrophils (Fig. **1E**).

### 3.2 Subtype Analysis of CRGs using Consistency Clustering

**Fig. (2):**
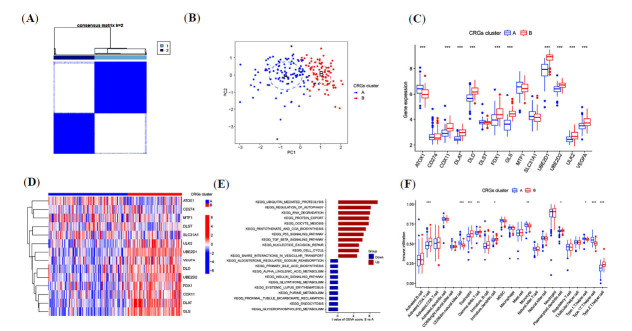
**CRG** expression-related subtypes and their biological characteristics based on CRGs expression, obtained through consistent clustering. (**A**) Defined consensus matrix heatmaps for the two clusters (k=2). (**B**) PCA showing significant differences between clusters A and B. (**C**) Heatmap showing the expression of 14 CRGs in the two CRG clusters. (**D**) Box plot depicting the expression of 14 CRGs within two CRG clusters. (**E**) GSVA of the biological pathways associated with the two subtypes. (**F**) Differences in the levels of infiltrating immune cells in the two CRG clusters. *P <0.05, **P < 0.01, ***P < 0.001.

Cluster analysis was performed on the differential expression of CRGs in sepsis-associated ALI patients to better understand the biological characteristics of CRGs. The consistency matrix heatmap indicated the optimal cutoff at K = 2 (Fig. **2A**). Two subgroups were defined, separating the sepsis-associated ALI patients into clusters A and B. The two distinct CRG clusters were separated on PCA (Fig. **2B**). Notably, COX11, DLAT, DLD, FDX1, GLS, UBE2D1, UBE2D2, ULK2, and VEGFA were downregulated in CRG cluster A, while ATOX1 was upregulated in this cluster (Fig. **2C**). The heatmap in Fig. (**2D**) illustrates the differences in CRG expression between the two subgroups. Furthermore, the GSVA analysis plot revealed the association of various pathways with the subgroups, where cluster B exhibited positive regulatory relationships with pathways such as ubiquitin-mediated proteolysis, regulation of autophagy, and RNA degradation. On the other hand, cluster A showed positive regulatory relationships with pathways like glycerophospholipid metabolism and endocytosis (Fig. **2E**). ssGSEA analysis demonstrated differences in immune cell infiltration between the two CRG clusters, with cluster B having higher levels of activated CD4+ T cells, eosinophils, γ, δ T cells, and Th2 cells compared to cluster A. Conversely, cluster A was enriched for CD56 natural killer cells, monocytes, and Th17 cells (Fig. **2F**).

### 3.3 Identification of Gene Modules and Co-expression Network Construction in the CRG Clusters

**Fig. (3):**
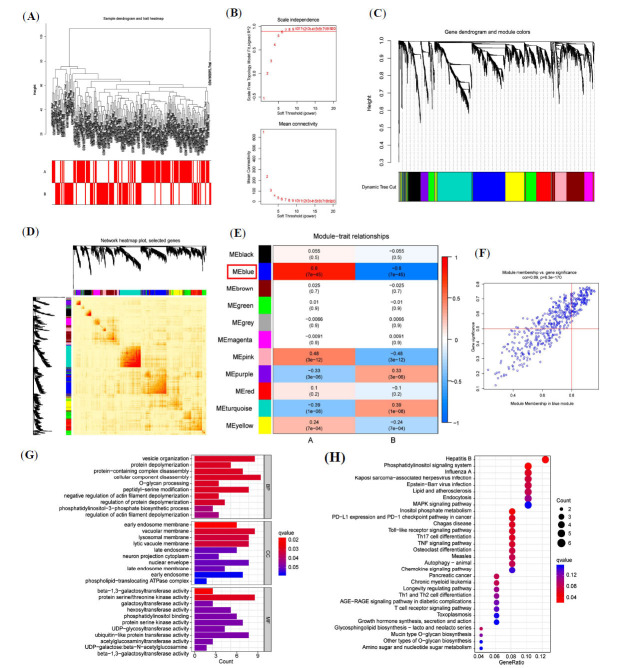
Construction of a gene co-expression network for the CRG clusters by WGCNA. (**A**) Weighted co-expression network of Cluster A and Cluster B. (**B**) Scale independence and mean connectivity of all genes. (**C**) Genetic dendrogram and module colors. (**D**) Heatmap showing visualization of the gene network. (**E**) Correlation analysis of the module signature genes with CRG clusters showing the highest correlation in the blue module. (**F**) Scatter plot of module membership and gene significance in the blue module. (**G**) GO analysis of hub genes associated with CRG clusters from three perspectives: biological processes, cellular composition, and molecular function. (**H**) KEGG pathway enrichment analysis of hub genes associated with CRG clusters.

To identify the hub gene modules associated with the CRG clusters, the WGCNA algorithm was utilized to construct co-expression networks and modules for both CRG subgroups. The biological functions of hub gene module enrichment were further analyzed. WGCNA first clustered the samples (Fig. **3A**), set the threshold to 2, and selected the top 20% of genes with the highest variance for further analysis (Fig. **3B**). The gene expression matrix was then identified in the individual modules, ensuring each module contained at least 100 genes (Fig. **3C**, **D**). Ultimately, a total of 11 co-expression modules were identified in the cohort (Fig. **3E**), with the blue module exhibiting the strongest positive correlation with Cluster A score (Cor=0.8, P=7e-45) and the strongest negative correlation with Cluster B score (Cor= -0.8, P=7e-45). In the blue module (Fig. **3F**), gene significance was set to >0.5 and gene-module correlation to >0.8, resulting in the identification of 132 hub genes associated with CRG clusters.

To further investigate the biological properties and the relevant activities and pathways in the CRG clusters, GO and KEGG pathway enrichment analyses were conducted on the hub genes (Fig. **3G**, **H**). The results indicated a significant enrichment in vesicle organization within the BP category, while the CC category showed pathways related to early endosomal membranes. MF terms included beta−1,3−galactosyltransferase activity, among others. KEGG analysis identified enriched pathways associated with hepatitis B, phosphatidylinositol signaling systems, and inositol phosphate metabolism.

### 3.4 Analysis of the Roles of the CRG-related Subgroups in Sepsis-associated ALI

**Fig. (4):**
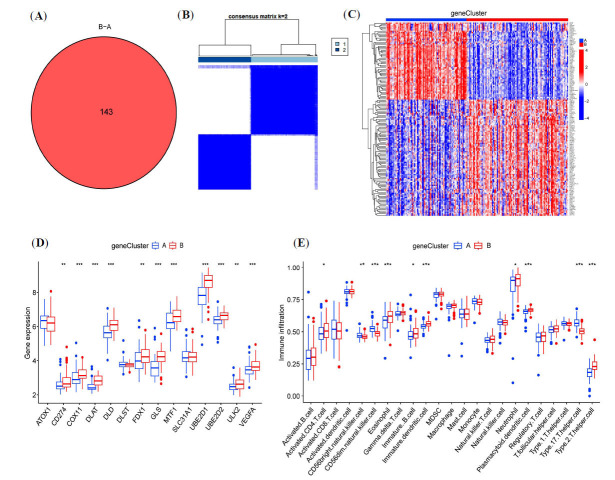
Cluster analysis of differentially expressed genes (DEGs) between CRG cluster A and cluster B to construct gene clusters. (**A**) A total of 143 DEGs were identified between the two CRG clusters. (**B**) Consensus clustering analysis of 143 genes at k = 2. (**C**) Heatmap of the distribution of 143 DEGs between the two gene clusters. (**D-E**) Differences in the expression of 14 CRGs and immune cell infiltration between the two gene clusters. *P <0.05, **P < 0.01, ***P < 0.001.

To further clarify the accuracy of the CRG cluster analysis, 143 CRG cluster-associated differentially expressed genes (DEGs) were identified (Fig. **4A**). Through the expression of DEGs, sepsis-associated ALI patients were categorized into gene cluster A and gene cluster B using a consensus clustering approach (Fig. **4B**). The heatmap in Fig. (**4C**) illustrates the differences in expression of the 143 DEGs between the two gene clusters, with 56 genes upregulated and 87 genes downregulated in gene cluster A. Fig. (**4D**) displays the differential expression of the two gene subgroups, aligning with the CRG subgroups. Fig. (**4E**) showcases the variation in immune cell infiltration between the two gene clusters, findings consistent with the CRG clusters.

**Fig. (5):**
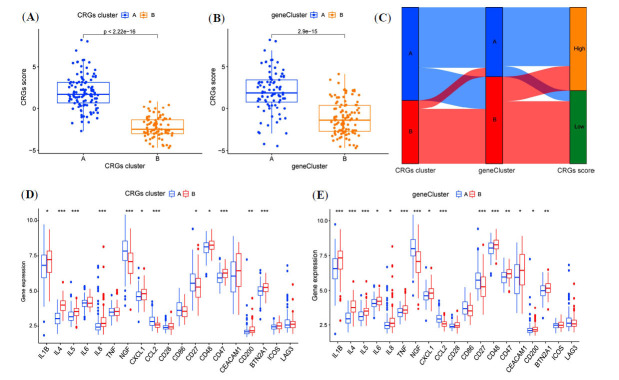
Characteristics of CRG subtypes in the identification of patients with sepsis-associated ALI. (**A-B**) Scoring of different gene clusters and CRG clusters. (**C**) The Sankey diagram illustrates the distribution among two CRG clusters, two gene clusters, and two CRG scoring groups. (**D-E**) Differential expression of immunomodulatory genes and immune checkpoints in different CRG clusters and gene clusters. *P <0.05, **P < 0.01, ***P < 0.001.

The comparison between the CRG clusters and gene clusters validated the accuracy of the cluster analysis of CRGs in patients with sepsis-associated ALI, revealing diverse expression patterns. The findings indicated that CRG cluster A had a significantly higher score than CRG cluster B, and gene cluster A scored significantly higher than gene cluster B (Fig. **5A**, **B**). Sankey plots illustrated the distribution of CRG scores, showing consistency between CRG cluster results with gene cluster results (Fig. **5C**). Given the crucial role of imbalances in inflammatory factors in the pathogenesis of sepsis-associated ALI, we further examined the differential expression of inflammatory factors and immune-related molecules in the CRG cluster and gene clusters (Fig. **5D**, **E**). These findings suggest that the two subgroups associated with CRGs may aid in predicting immune response patterns in patients with sepsis-associated ALI.

### 3.5 Construction and Validation of the CRG Predictive Model

**Fig. (6):**
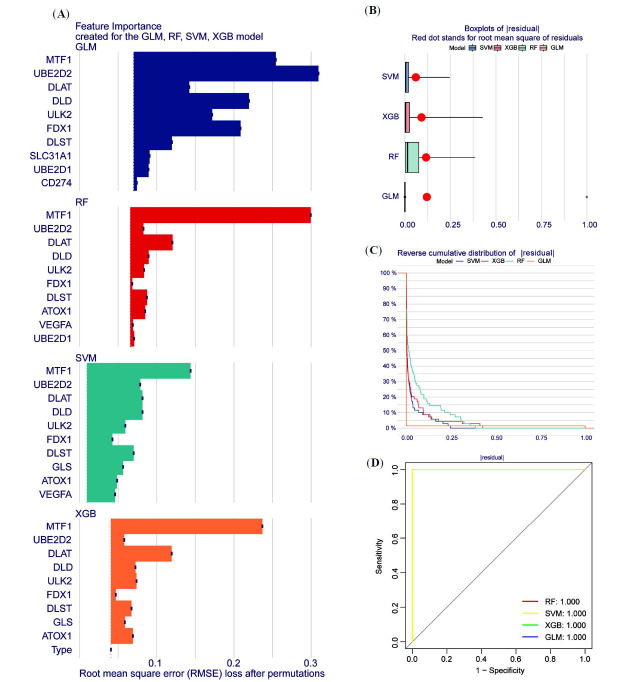
Construction and evaluation of machine learning models. (**A**) Importance scores for machine models. (**B**) RF, SVM, GLM, and XGB residual box plots. (**C**) Plot of the reverse cumulative distribution of residuals. (**D**) The accuracy of the four machine learning models was evaluated using ROC curves.

**Fig. (7):**
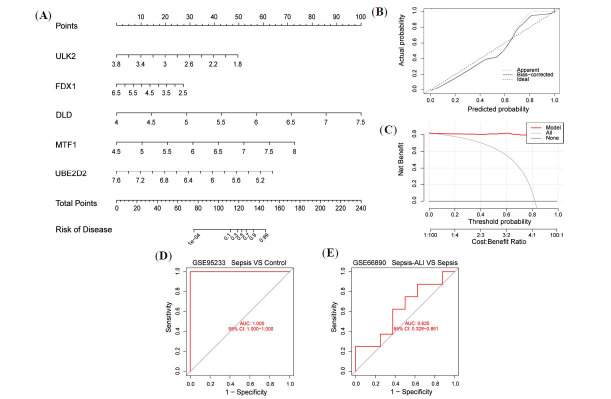
A predictive model for sepsis-associated ALI based on the expression of CRGs. (**A**) Predictive nomogram constructed based on the expression of the top 5 CRGs in the GLM model. (**B**) Verify the predictive ability of the nomogram through calibration curves. (**C**) Testing the accuracy of the model with decision curves. (**D-E**) ROC curves using the GSE95233 and GSE66890 datasets to validate the value of nomograms for the assessment of sepsis-associated ALI.

To further understand the characteristics of the diagnostic CRGs, four validated machine learning models were built, including RF, SVM, GLM, and XGB. In the plot showing the relative importance (Fig. **6A**), the GLM model has the highest score. In the box line plot of the residuals for the model (Fig. **6B**), the red dots represent the root mean square of the residuals, with the smallest residuals seen in the GLM model. The conclusion drawn from the residual reverse cumulative distribution diagram is consistent with the above results (Fig. **6C**). The ROC curve results revealed that all four models had an area under the curve (AUC) of 1. From the combined results, it was concluded that the GLM model had the highest prediction accuracy (Fig. **6D**). The top five gene variables (MTF1, UBE2D2, DLAT, DLD, and ULK2) were selected as disease signature genes in the GLM model to construct a nomogram for predicting the incidence of sepsis-associated ALI (Fig. **7A**). Scoring intervals were established for each trait gene, and their cumulative scores were utilized to determine the relative incidence rate. The solid and dashed lines in the calibration curve plot are closer together (Fig. **7B**), and the red line in the decision curve plot is further away from the other curves (Fig. **7C**), both highlighting the superior accuracy of the model. Finally, validation of the CRGs model was performed using two datasets, GSE95233 and GSE66890, confirming the model's efficacy in predicting sepsis and sepsis-associated ALI incidence (Fig. **7D**, **E**).

### 3.6 CRG Expression in Human Alveolar Epithelial Cells after LPS Treatment

**Fig. (8):**
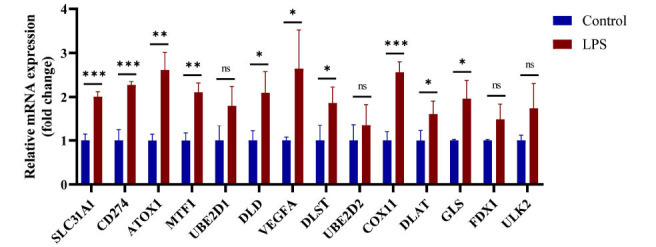
Expression of CRGs in a sepsis-associated ALI cell model was detected by qPCR. *P<0.05,**P<0.01, ***P<0.001.

Damage to the alveolar epithelium plays a crucial role in the pathogenesis of sepsis-associated ALI. We established an *in vitro* cellular model of sepsis-associated ALI using A549 human alveolar epithelial cells treated with LPS. Differential expression of CRGs was observed both before and after treatment (Fig. **8**). Our results indicated an upregulation of SLC31A1, CD274, ATOX1, MTF1, DLD, VEGFA, DLST, COX11, DLAT, and GLS, which aligns with previous bioinformatics analysis and the expression profile of CRG cluster B. These findings suggest the involvement of CRGs in the immune-mediated inflammatory response of sepsis-associated ALI.

## 4 DISCUSSION

Pathogenic infections in sepsis can cause severe inflammatory damage to the lungs, leading to ALI/ARDS in critically ill patients [36]. The immune response plays a crucial role in the development of sepsis-associated ALI, with irreversible ALI potentially leading to fatality in severe cases [37]. Cuproptosis is a copper-dependent form of cell death associated with mitochondrial dysfunction [38]. Systematic bioinformatics approaches have been developed to explore the relationship between cuproptosis and non-neoplastic diseases [17]. Studies have shown that cuproptosis-related regulators are closely linked to immune cells, immune function, and drug effectiveness [39]. The specific roles of cuproptosis and cuproptosis-based markers in sepsis-associated ALI remain unclear, and further research is necessary.

The pathogenesis of sepsis-associated ALI is multifaceted and believed to involve cytotoxic mechanisms [40]. Gene expression profiling has revealed that ferroptosis, pyroptosis, apoptosis, ferroptosis, and cuproptosis play significant roles in the development of organ dysfunction in sepsis [41-43]. The cuproptosis pathway provides a promising approach for sepsis treatment [44]. Recent findings suggest that cuproptosis can enhance the effectiveness of immune checkpoint inhibitor-based immunotherapies [45]. Nanosystems designed to induce cuproptosis have been developed as drug delivery platforms for anti-tumor and anti-microbial therapies [46]. Copper complex nanoparticles have shown efficient delivery of copper complex into cells to trigger cuproptosis [47]. The accumulation of metal ions can result in oxidative stress within cells, leading to cellular damage [48]. Nanoparticles combining drugs and copper have demonstrated the ability to induce cuproptosis in cancer cells and stimulate immune responses, offering novel strategies for disease treatment [49]. The relationship between CRGs and immune regulation and metabolism in sepsis-associated ALI presents innovative avenues for therapeutic interventions through the cuproptosis pathway.

Gene microarray datasets based on clinical samples are crucial for assessing sepsis and other challenging inflammatory conditions [50, 51]. This study represents the first comprehensive analysis of changes in and interactions of CRGs at the transcriptional level in sepsis-associated ALI. Fourteen CRGs were validated in patients with sepsis-associated ALI, and their role in disease progression was systematically examined. Significant differences in immune cell infiltration were observed between controls and sepsis-associated ALI patients, with most CRGs showing correlations with immune cells. The lung is an important immune organ housing both innate and adaptive immune cells, relying on alveolar macrophages, innate lymphoid-like cells, and various pattern recognition receptors in the context of sepsis-associated ALI [37]. During sepsis, proinflammatory mediators released by alveolar macrophages exacerbate ALI severity by promoting neutrophil infiltration into the lung [52]. It is anticipated that future research will increasingly explore the relationship between epigenetics and immune regulation in sepsis-associated ALI.

To further investigate the roles of CRGs and their correlation with immune cell infiltration, patients with sepsis-associated ALI were classified based on CRG expression. Identifying distinct subtypes of sepsis-associated ALI at a molecular level could facilitate the development and application of appropriate treatment regimens for patients with varying subtypes [53]. Through unsupervised clustering analysis, patients were categorized into two CRG clusters and two gene clusters. Interestingly, both the CRG and gene clusters exhibited similar patterns in immune cell abundance and showed comparable PCA scoring results, which helped to predict the immune characteristics of sepsis-associated ALI. Activation of intracellular immune response pathways plays a crucial role in disease progression [54]. The acute response of the host to invasive pathogens usually activates immune cells, leading to the production of multiple proinflammatory mediators and triggering a cytokine storm [55]. Both CRG and gene clusters showed similar trends in the levels of inflammation-related factors. These CRGs encode essential intracellular regulatory molecules involved in cuproptosis and can serve as biomarkers for inflammatory diseases, participating in intracellular signaling and modulating inflammatory responses [56, 57]. UBE2D1 has been identified as a potential biomarker for diabetes-related sepsis [58]. In a study by Lu et al., miR-522-3p was found to inhibit T cell proliferation and activation by targeting SLC31A1 expression [59]. Copper supplementation enhanced CD274 expression at both the mRNA and protein levels [60]. The CD274/PD-L1 gene is a key molecule in the immune checkpoint machinery and has been widely used in immunotherapy to treat different diseases [61]. Upregulation of miR-10a-3p was observed to downregulate COX11 and activate the NF-κB signaling pathway, promoting the progression of mycoplasma pneumonia [62].

Copper homeostasis is regulated by various processes, including absorption by cells and tissues, circulation in the blood, and tissue utilization [63]. We searched for phenotypically relevant biological functions of CRG clusters using WGCNA. Research has shown that copper's involvement in secretory pathways can be initiated by the activation of PI3K/Akt intracellular signaling [64], aligning with our own findings. Further exploration of the metabolic pathways associated with CRG subtypes using GSVA analysis revealed a significant connection to autophagy regulation. Autophagy degrades damaged organelles and proteins, and autophagy gene polymorphisms are associated with the development of inflammatory lesions [65]. These genes are crucial in a variety of cellular processes, including mechanistic pathways related to cuproptosis [66]. Molecular pathways regulated by cuproptosis also influence cellular autophagy, contributing to disease progression [67]. DLAT plays a vital role in mitochondrial function by encoding the pyruvate dehydrogenase complex [17, 68]. Studies have shown that SLC31A1-dependent copper deficiency promotes autophagy, acting as a protective mechanism against cell death in copper deficiency [69]. Chen *et al.* revealed that CRIP2 interacts with ATOX1 in the nucleus, with ATOX1 transferring copper to CRIP2, facilitating ubiquitin-mediated proteasomal degradation and involvement in the activation of autophagy [70].

Four prediction models based on the expression levels of CRGs were compared, with the GLM model demonstrating significantly higher accuracy. A nomogram was developed using the top five CRGs with the highest scores in the GLM model to predict the risk of sepsis-associated ALI. The total gene scores in the nomogram can accurately predict the risk of sepsis-associated ALI and have been linked to acute inflammatory diseases. Research has shown that deletion of MBD2 affects intracellular zinc homeostasis by upregulating MTF1, thereby reducing LPS-induced apoptosis and ALI in mouse alveolar epithelial cells [71]. Circ_UBE2D2 has been found to target binding sites for multiple miRNAs associated with septic acute kidney injury, impacting disease progression [72]. ULK2 plays a tissue-specific role in regulating autophagy and other cellular processes [73]. Recent studies have highlighted the co-expression of DLAT with immunostimulatory genes, immune inhibitors, chemokines, and chemokine receptors [74]. Both DLD and DLAT have been proposed as prognostic biomarkers and potential targets for cancer immunotherapy [74, 75]. The ROC and calibration curves demonstrated the significant predictive performance of the nomogram, suggesting that this quantitative signature could enhance diagnostic assessment and personalized patient treatment.

We utilized a sepsis-associated ALI cell model to validate whether the alterations in CRGs were consistent with the results of the bioinformatics analysis. The qPCR results indicated that the expression pattern of CRGs resembled that of CRG cluster B, suggesting that the cellular biological processes might be akin to those observed in cluster B. However, differences exist between the cell model and the human sample, and the samples in the dataset come from the patient's peripheral blood. The changes in CRG expression demonstrate the significance of these genes in the progression of sepsis-associated ALI and are worth further research. Identifying crucial genes is vital for disease diagnosis and treatment, and delving into the molecular mechanisms of disease development can facilitate the development of novel therapeutics [76]. CRGs as a nascent research domain, examining the association and diagnostic utility of CRGs in sepsis-associated ALI could pave the way for more molecular investigations.

There are some limitations in this study, including its reliance on bioinformatics analysis without extensive clinical data and validation experiments. Public databases provide single regional datasets, limiting diversity in patient ethnicity representation. The predictive value of CRG scores requires validation in large clinical cohorts. Despite few enriched pathways in GO and KEGG analyses due to the small sample size, these pathways are linked to the CRG phenotype, reinforcing the study's findings.

## CONCLUSION

In summary, this study is the first to explore and elucidate the potential regulatory mechanisms of CRGs in sepsis-associated ALI. It analyzed the connections between CRGs, immune cell infiltration, and the levels of inflammatory and immunoregulatory molecules in sepsis-associated ALI. Additionally, a prediction model was developed to assess the predictive capability of CRGs for the risk of sepsis-associated ALI. These findings provide new clues for investigating the pathogenesis and treatment of sepsis-associated ALI.

## Figures and Tables

**Table 1:** Primer sequences of 14 CRGs.

**Gene**	**Primer Sequence**	
	**Forward**	**Reverse**
ATOX1	5'-TCATGCCGAAGCACGAGTT-3'	5'-CTTCAGGGTTGCAAGCAGAG-3'
CD274	5'-AGAACTACCTCTGGCACATCCTC-3'	5'-AACGGAAGATGAATGTCAGTGCTA-3'
COX11	5'-GCAGAACAAGACGACCCTCACT-3'	5'-GCTGATCCTCCAAGTCCAGTAGTC-3'
DLAT	5'-CCTCCCACAGGTCCTGGAAT-3'	5'-GTGCAATAACCCGACGAATGT-3'
DLD	5'-CTGGCTCACAAAGCAGAGGAT-3'	5'-GCTGTTAGCAGCAAATGGGAA-3'
DLST	5'-CTAACAGCAGGAAGGTTGTCATT-3'	5'-CCACCTGACATCTCCCTCTGT-3'
FDX1	5'-CCACTTTATAAACCGTGATGGTG-3'	5'-ACATGCACCAAAGCCATCAA-3'
GLS	5'-ACTTCTCAGGGCAGTTTGCTTTC-3'	5'-TATCCAGAGGAGGAGACCAGCAC-3'
MTF1	5'-GTGACTTGAGCCTTCTGTCCAC-3'	5'-TGAAATTGTGCTGAGGTCCTG-3'
SLC31A1	5'-TACAATTCCATGCCTGTCCCA-3'	5'-GCTTATGACCACCTGGATGATGT-3'
UBE2D1	5'-GGTCACCAGCTCTGACTGTAT-3'	5'-CTGAGTCCATTCTCTTGCATGT-3'
UBE2D2	5'-AGAGAATCCACAAGGAATTGAATGA-3'	5'-TAGGGACTGTCATTTGGCCC-3'
H-ULK2	5'-TCAAGCATCTTCCAACCTGTTAG-3'	5'-TAAACTGTCTGTGCTGCCCTGAT-3'
VEGFA	5'-CCCACTGAGGAGTCCAACATC-3'	5'-TACACGCTCCAGGACTTATACCG-3'

## Data Availability

This study is an analysis of publicly available datasets, all of which can be found in the GEO database(https://www.ncbi.nlm.nih.gov/gds/).

## LIST OF ABBREVIATIONS

ALI: Acute Lung Injury
GEO: Gene Expression Omnibus
CRGs: Cuproptosis-related Genes
ARDS: Acute Respiratory Distress Syndrome
TCA: Tricarboxylic Acid
PCA: Principal Component Analysis
GSVA: Gene Set Variation Analysis
WGCNA: Weighted Gene Co-expression Network Analysis
TOM: Topological Overlap Matrix
GO: Gene Ontology
KEGG: Kyoto Encyclopedia of Gene and Genomes
BP: Biological Processes
CC: Cellular Components
MF: Molecular Functions
GLM: Generalized Linear Model
RF: Random Forest
SVM: Support Vector Machine
XGB: Extreme Gradient Boosting
ROC: Receiver Operating Characteristics
LPS: Lipopolysaccharide
AUC: Area Under the Curve
